# Analytical method to measure three-dimensional strain patterns in the left ventricle from single slice displacement data

**DOI:** 10.1186/1532-429X-12-33

**Published:** 2010-06-01

**Authors:** Abbas Nasiraei Moghaddam, Nikoo R Saber, Han Wen, J Paul Finn, Daniel B Ennis, Morteza Gharib

**Affiliations:** 1 Department of Radiological Sciences, Diagnostic Cardiovascular Imaging Section, David Geffen School of Medicine, University of California, Los Angeles, CA, USA; 2 Bioengineering Option, California Institute of Technology, Pasadena, CA, USA; 3 National Heart, Lung, and Blood Institute, National Institutes of Health, Bethesda, MD, USA

## Abstract

**Background:**

Displacement encoded Cardiovascular MR (CMR) can provide high spatial resolution measurements of three-dimensional (3D) Lagrangian displacement. Spatial gradients of the Lagrangian displacement field are used to measure regional myocardial strain. In general, adjacent parallel slices are needed in order to calculate the spatial gradient in the through-slice direction. This necessitates the acquisition of additional data and prolongs the scan time. The goal of this study is to define an analytic solution that supports the reconstruction of the out-of-plane components of the Lagrangian strain tensor in addition to the in-plane components from a single-slice displacement CMR dataset with high spatio-temporal resolution. The technique assumes incompressibility of the myocardium as a physical constraint.

**Results:**

The feasibility of the method is demonstrated in a healthy human subject and the results are compared to those of other studies. The proposed method was validated with simulated data and strain estimates from experimentally measured DENSE data, which were compared to the strain calculation from a conventional two-slice acquisition.

**Conclusion:**

This analytical method reduces the need to acquire data from adjacent slices when calculating regional Lagrangian strains and can effectively reduce the long scan time by a factor of two.

## Introduction

The left ventricular deformation associated with the pumping of blood from the heart is known to be complex [1]. For example, it is known that there are significant regional variations in the time course, magnitude, and spatial pattern of deformation within the ventricular wall [2]. Moreover, in the course of heart failure and other cardiac diseases, wall motion abnormalities become very pronounced and deviate from the normal, healthy function of the myocardium. Therefore, it is of significant clinical interest to quantify these regional wall motion abnormalities with high temporal and spatial resolution and therefore derive indices of the nature and extent of cardiac dysfunction to better inform clinical decision-making.

Cardiovascular magnetic resonance (CMR) motion encoding strategies have emerged in recent years as clinically useful tools for assessing derangements in regional cardiac function. In particular, CMR phase contrast (PC) [3,4], tissue tagging [5,6], HARP [7], and DENSE [8] have arisen as powerful techniques for encoding cardiac motion in a CMR exam and each has specific advantages and disadvantages. DENSE displacement encoding is similar in principle to the PC velocity-encoding method in that the motion components of small tissue volumes are encoded in the phase image at every image pixel location [9]; hence, the number of measures across the LV wall is typically higher than the number of tags across the wall in a tagging experiment. As an example, DENSE has been used successfully for detecting small focal regions of abnormal contraction in patients [10], in characterizing mouse ischemia models [11], and in acquiring detailed functional data in canine hearts *in vivo *[12].

The DENSE method has been implemented in different ways. In the meta-DENSE technique, which is used for data acquisition in this study, a series of phase-labeled images are acquired at the same time point in the cardiac cycle. The tissue can be phase-labeled at different points in the cardiac cycle prior to the image acquisition. The result is that the same pixel location in each image always corresponds to the same physical tissue. This ensures that the behavior (displacement and deformation) of the same "particles" within the tissue are followed within the frame of reference. This is oftentimes referred to as the "Lagrangian" framework for "tissue tracking." Kim et al. have proposed a variation of the DENSE sequence for two-dimensional (2D) breath-hold cine DENSE imaging [13] which was later extended to 3D by using the slice following technique [14]. Compared to meta-DENSE, cine DENSE acquires the data faster but with lower signal-to-noise ratio (SNR) and not in a Lagrangian framework.

The three-dimensional (3D) principal shortening strain is typically not oriented in any standard short or long axis imaging plane, therefore 2D measures typically underestimate the maximal shortening undergone at any point. From the 3D Lagrangian displacement vector for each pixel in the image, we are able to calculate the Lagrangian strain, which provides a complete description of myocardial deformation. The Lagrangian strain is calculated from the spatial gradients of the displacement vector field. In general, this requires that there exist two or more displacement vectors along each of the orthogonal directions along which the gradients will be computed. With typical 2D images, however, there are insufficient displacement data in the through-plane direction and calculation of the full 3D strain tensor is not possible unless we acquire displacement data in orthogonal directions or parallel planes. Acquiring multiple planes of data, however, increases the number breath holds, thereby extending the exam duration. The analysis can also be complicated in multiple plane acquisition because of image misregistration [15].

Several efforts have sought to make 3D strain calculation more efficient. Osman et al. acquired the out-of-plane strain using SENC [16], which derives the through-plane strain from the frequency of tag planes applied parallel to and within the image plane. Abd-Elmoniem et al. proposed zHARP [17], which is a modified version of slice following-CSPAMM [18,19] and uses HARP processing to provide 3D displacement in a Lagrangian frame. They recently used "stacks of zHARP images" to derive the 3D strain tensor [20]. Hess et al. proposed a method that utilizes 2D DENSE in-plane displacement measurements in conjunction with a SENC through-plane strain measure [21]. These techniques are based on two or more slices and require at least 4 breathhold acquisitions. Therefore, they suffer from inter-breathhold myocardial position variability, which can cause a significant increase in the strain error (RMS error of 0.04 in through-plane normal strain) [21]. Sampath et al. presented a combination of HARP and SENC for 3D strain measurement [22]. The latter is the only technique that requires no more than one image slice for strain measurement; however, it does not calculate the complete 3D strain tensor because the shear components are not measured.

The goal of this work was to define the mathematics for calculating all components of the 3D strain tensor from a single slice of 3D displacement data by assuming tissue incompressibility. The importance of calculating out-of-plane strains from a single slice is emphasized by noting that in many strain imaging techniques (such as zHARP, slice following 3D cine DENSE and DENSE-SENC), the distance between adjacent slices may be too large to accurately calculate the through-plane strains due to the curvature of the heart in the through-plane direction. Herein, we derive a mathematical approach and the image processing tools to increase the derived 3D deformation information acquired from a DENSE CMR exam. Our methods are sufficiently general and can easily be extended to other 3D deformation imaging methods (e. g. zHARP). Specifically, we calculate the out-of-plane components of the Lagrangian strain tensor in addition to measuring the in-plane components from a single-slice DENSE CMR dataset covering a complete cardiac cycle.

The method has been validated using a simulated data set, which has an analytical deformation gradient tensor and mimics the incompressible deformation of the moving myocardium. It has also been compared to conventional methods that use two adjacent slices for strain measurements. Finally, we demonstrate the feasibility of the method in a human subject and conclude by comparing our results to those of other studies.

## Theory

### Deformation gradient tensor

The displacement data is amenable to analysis by continuum mechanics methods. The deformation of a continuum is described by the second order deformation gradient tensor ***F***, which is defined as the partial differentiation of the spatial position vector with respect to the reference (material) coordinates. The positions of the material points in the reference (undeformed) and deformed configurations are *X *and *x*, respectively. These measures are related as follows:(1)

Therein ***F ***is the deformation gradient tensor and *dX *is the position vector difference between material points (distance vector) in the reference frame, which is transformed to *dx *in the deformed frame. Each element of this tensor (*F*_*ij*_) is defined as ∂*x*_*i*_/∂*X*_*j *_and determines how the distance between any two particles along the *j*th dimension in the reference configuration is projected to the *i*th direction in the deformed configuration. This linear relation is determined separately at each material point to describe the deformation of all displacement vectors included in a small neighborhood (an infinitesimal volume). Beyond this infinitesimal volume, Equation 1 becomes an approximation for actual measurable distances. Once ***F ***is computed, the Lagrangian strain tensor ***E ***can be calculated accordingly:(2)

where *I *is the identity tensor. Tensor ***F ***is calculated in the Cartesian coordinate system and is subsequently transformed to a locally polar coordinate system, which is more popular in cardiac strain measurement, accounting for the elongated ellipsoid shape of the left ventricle. The three unit vectors of this system lie in the local Radial, Circumferential, and Longitudinal (RCL) directions at any given point of the heart. Once the long axis of the heart and the local radial direction are known, these three directions can be defined uniquely for any point within the myocardium, including those on the epicardium and endocardium. These directions can be robustly calculated almost everywhere in the myocardium, apart from near the apex where they are not well defined (e. g. the radial and longitudinal directions become coaxial).

### Displacement smoothing

The elements of the deformation gradient tensor are calculated using spatial derivatives (*F*_*ij *_= ∂*x*_*i*_/∂*X*_*j*_), which is a noise sensitive operator. Therefore, the spatial and temporal smoothing of the measured displacement should be applied prior to taking the derivative. Previously, it has been shown that the significant temporal frequencies of the heart motion are lower than five times the heart rate [23]. Hence, a fifth order Fourier basis function should be sufficient for temporal smoothing.

Noise can induce outlier displacements in the tissue tracking process, especially in regions of low signal intensity where phase measurements are especially sensitive to noise. These outliers can generate large errors in the calculation of the local strain. Median filters are quite popular and effective for suppressing this type of noise [24]. To decrease the possible adverse effects of filtering on the strain calculation, the smallest symmetric 2D median filter, which is the five-pixel plus sign-shaped filter, was applied to each component of the displacement vector to reduce the effect of the outliers. This is the minimum amount of smoothing that can be obtained with a median filter. For a displacement field with a higher noise level, however, we may need to increase the size of the applied median filter. Although components of ***F ***relating to the out-of-plane direction are more noise-sensitive, no further filtering or smoothing was applied to the displacement in the out-of-plane direction. Instead, we invoked specific mathematical methods to calculate individual elements of the ***F ***tensor, which determine the out-of-plane deformation.

### Estimation of the deformation gradient tensor

As stated in definition of the deformation gradient tensor, Equation 1 is only true for an infinitesimal local neighborhood. For actual measurable distances, however, the first order linear Equation 1 is not sufficient, in general, to relate distance vectors before and after deformation and thus a residual error can result from this linear approximation. It is worth noting that the residual error caused by points from adjacent slices is more severe, since the distance between adjacent slices is significantly greater than the in-plane distances. The error in the calculated deformation gradient tensor may also increase as a consequence of the noise in the data. To decrease the sensitivity of the calculation to noise, therefore, it is judicious to estimate the nine elements of tensor ***F ***over a neighborhood that consists of more than nine data points and approximate the solution of this over-determined set of equations through a linear least-square approach that minimizes the residual error [25]. The extent of tissue area covered by this neighborhood in the imaging plane is called the smoothing area. The estimation of ***F ***is aimed at minimizing the L_2 _distance of the error, i.e. the following quantity *L*, which is the sum of squared residuals over its smoothing area.(3)

It is important to choose a suitable region for the smoothing area. A large value for the smoothing area will over-smooth the physiologic spatial variations in elements of ***F ***rather than merely reducing the noise, whereas a small smoothing area does not reduce the noise sufficiently. This matter is discussed in more detail in the Discussion section.

In the conventional method for strain estimation, based on two or more adjacent slices, an equivalent procedure is followed, but points are chosen from both adjacent planes. With one slice, however, the small spatial variation of the data points in the direction normal to the surface nearly imposes a singularity condition, which makes the gradients in that direction highly sensitive to noise. It requires, therefore, exploiting some physical/mathematical constraints. This singularity condition does not exist when two adjacent slices are employed for strain measurement in the conventional methods.

To improve the accuracy of our calculation based on a single slice data, we compute the direction normal to the imaged surface for each neighborhood and redefine a local coordinate system using this normal vector as the out-of-plane unit vector. Calculation of ***F ***in this new coordinate system ensures that the effect of the singularity condition only appears in the third column of the deformation matrix. It also implies that the expected error in the calculation of the third column of ***F ***is greater than the expected error in the first two columns of ***F***. Recall that ***F ***is not symmetric and since the out-of-plane motion is measured by meta-DENSE with similar precision to the in-plane components, the calculation of the third row of ***F ***is as well-posed as its first two rows. In order to compute the third column (out-of-plane elements) of ***F ***with reduced noise sensitivity, we employed the following mathematical methods and physical assumptions.

#### 1) Incompressibility of the myocardium

Myocardial tissue volume may vary over the cardiac cycle due to blood volume changes. However, measurements in canine hearts *in vivo *show that the level of volume fluctuation is approximately 1% [26]. Therefore, tissue incompressibility is a reasonable assumption in the heart. The deformation gradient tensor for an incompressible body has a determinant equal to unity. This extra equation increases the robustness in calculation of the third column of ***F ***and was enforced in all of our calculations.

#### 2) Combining *F *with the inverse-deformation gradient tensor *G*

***F ***is the tensor that transforms the reference positions of the myocardial points within a neighborhood (smoothing area) to an arrangement, as close as possible, to their deformed shape. It is also possible to simultaneously define the tensor ***G ***to perform the inverse operation. ***G ***maps the same set of points in the deformed shape to an arrangement, as close as possible, to their reference position. Ideally, the ***G ***tensor should be the inverse of the ***F ***tensor, but this is not actually the case, since we can only calculate a least-square estimate for each of these tensors. In fact, neither of the matrices performs the perfect matching of original and deformed points as a consequence of least-squares estimation.

The strain calculation can be improved by correcting the out-of-plane deformation and strains based on a combination of ***F ***and ***G***. The *F*_*33 *_element of the ***F ***tensor can be easily estimated based on in-plane elements of tensor ***G***, recalling the incompressibility of the myocardium (det(***G***) = 1):(4)

Furthermore, the orthogonality of the columns of ***F ***to the rows of ***G***, aids the recalculation of *F*_*13 *_and *F*_*23*_. Therefore, for *F*_*13 *_we have:(5)

**Figure 1:**
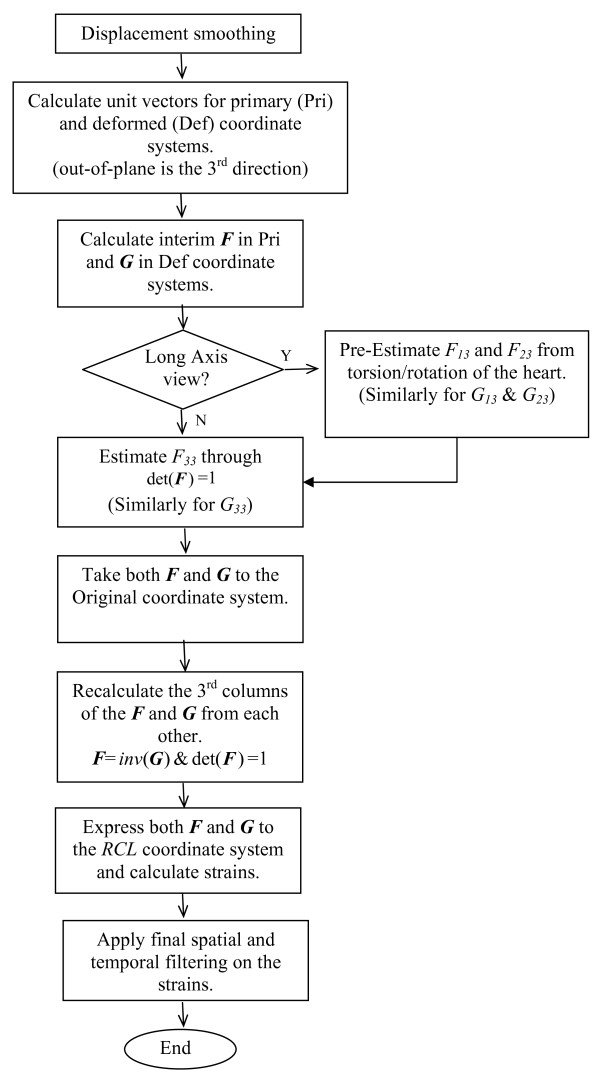
**The flowchart of the overall algorithm for estimation of deformation gradient tensor elements**.

The flowchart of the overall algorithm is shown in Figure 1. This algorithm results in a reasonable expected error for estimation of the deformation gradient tensor as described in Appendix A.

##### *E*_*33 *_*Correction*

*E*_*33 *_is calculated only from the third column of ***F***, which contains the elements of the deformation gradient tensor that are most sensitive to noise:(6)

As a consequence, the estimated error in *E*_*33 *_is more than two times greater than the error in the in-plane strains elements, which results in a rather significant relative error. An alternative way to estimate *E*_*33*_, the out-of-plane strain, is through approximating the thickening of the layer. By considering the problem in 2D and projecting the points on the image plane, each voxel is thereby represented by a small quadrilateral on that plane. We use the in-plane elements of the deformation gradient tensor to determine the changes in the area of this quadrilateral. Calling this area *S*_*1 *_and *S*_*2 *_before and after deformation, we have:(6a)

where *λ*_1 _and *λ*_2 _are stretch ratios of the 2D deformation in the principal directions. *λ*_1 _and *λ*_2 _are calculated as the square roots of the eigenvalues of the corresponding right Cauchy-Green deformation gradient tensor ***C ***= ***F***^*T*^***F ***in 2D (in-plane deformation). By accounting for the incompressibility of the myocardium, the stretch in the third (out-of-plane) direction is estimated as  and, for relatively small deformations, the corresponding strain is estimated as . To minimize the noise, we use both matrices ***F ***and ***G ***in this calculation. Considering the reverse action of the inverse deformation gradient tensor ***G***, *E*_*33 *_is estimated from the following equation:(7)

where λ_*iF *_and λ_*iG *_are the stretch ratios derived from ***F ***and ***G ***when they are reduced to a 2 × 2 (in-plane) matrix.

#### 3) Semi-symmetric rotation in the long axis view

**Figure 2:**
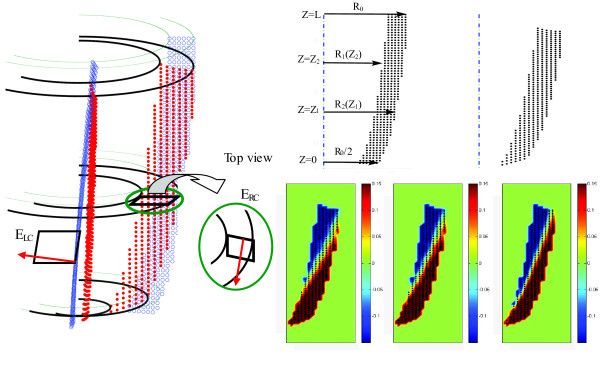
**(Left) Estimation of F_13 _and F_23 _using the torsion of the heart as the main factor in generating the shear strain components**. Blue and red dots show the position of the myocardium before and after deformation respectively. The red vector represents the out-of-plane direction. (Right top) The geometry of the LV and related parameters are shown in the simulated data before and after deformation. R_1 _and R_2 _are the internal and external radius of the LV respectively and Z shows the longitudinal access. In this model, at Z = 0 the external radius, R_2_, is reduced to half compared to its value at Z = L, R_0_. The thickness of the myocardium is modeled as R_0_/4 everywhere prior to deformation. Detailed formulation of geometry and deformation are mentioned in Appendix B. (Right bottom) Three mappings of element *F*_*21 *_of the deformation gradient tensor ***F ***at t = 390 ms in the simulated data. *F*_*21 *_is calculated from the proposed algorithm with a 25 mm^2 ^neighborhood (left map); the proposed algorithm with a 15 mm^2 ^neighborhood (middle map); and the analytical calculations (right map). Despite similar patterns, the fast spatial changes in this quantity caused a relative RMS error of 33.5% and 26.1% in (left) and (middle) respectively, caused by smoothing.

The torsional motion of the LV around the long-axis is almost independent of the azimuthal angle, which points along the circumferential direction. Herein, this characteristic of the LV is called the semi-symmetric rotation. Elements *F*_*13 *_and *F*_*23 *_of the deformation gradient tensor describe how the distance between two points along the out-of-plane (approximately circumferential) direction in the reference configuration is related to each of the in-plane distances (approximately radial and longitudinal) in the deformed configuration. This relation is closely associated with the torsional shear as illustrated in Figure 2. Therefore, when studying long-axis images, these two elements (*F*_*13 *_and *F*_*23*_) can be pre-estimated from the torsional motion of the heart, which is assumed to be a semi-symmetric rotation. Appendix B explains how under this condition the effect of rotation in the deformation tensor can be isolated and then estimated from the torsional movement of the heart, which is assessed locally from DENSE data. As a matter of fact, this torsion is the main factor in generating the shear strain components associated with the circumferential direction. This pre-estimation is useful for more accurate estimation of other elements of ***F ***and ***G ***according to Equation 5. Yet they will be modified later according to the algorithm shown in Figure 1.

In the short axis view, the effect of left ventricular rotation can be calculated with high precision, eliminating the need for a symmetry assumption. Left ventricular torsion, however, cannot be approximated and used for calculation of longitudinally associated shear strains.

## Methods

### DENSE CMR data acquisition

In this study, we used a specific version of DENSE CMR sequence called meta-DENSE [27]. DENSE CMR images were acquired on a three-Tesla (3T) whole body CMR scanner (Siemens Trio, Erlangen, Germany) at the Caltech Brain Imaging Center with an eight-channel cardiac array coil. Our study was approved by the institutional review board, and informed consent was obtained. A single-slice long axis dataset of high spatial and temporal resolution was acquired from the heart of a healthy 25-year-old female volunteer. The experiment was carried out under free-breathing conditions over a period of 40 minutes, which is directly proportional to the number of phases acquired (20 phases for this study). To synchronize the pulse sequence with the motion of the chest and heart, respiratory and heart monitoring were achieved using a pneumatic bellows and ECG monitoring.

In order to acquire directly the Lagrangian displacement vectors relative to a fixed time during the cardiac cycle, the image acquisition section of the DENSE sequence was positioned at a constant time in the late diastolic phase of the cardiac cycle when the myocardium is almost at rest. This fixed time was determined as 715 ms after the R-wave of the ECG trigger for this volunteer, while the encoding section was placed at a series of time steps in 30 ms increments after the ECG trigger.

The imaging parameters included FOV = 192 mm × 108 mm; acquisition matrix size = 128 × 72 pixels, pixel size = 1.5 mm × 1.5 mm; prescribed slice thickness = 5 mm; repetition time (TR) = 3.1 ms; mixing time (TM) = 600 ms; number of averages = 3; the displacement encoding sensitivity (k_e_) = 0.80 and 0.93 cycle/cm for in-plane and out-of-plane directions respectively. 20 phases were acquired with increasing delays of 30 ms increments after the trigger, which together covered 600 ms of the cardiac cycle starting 30 ms after detection of the QRS complex and ending during diastasis (acquisition window = 850 ms). In this manner, the time-resolved deformations from the beginning of systole throughout most of diastole were covered. The heart is almost at rest during the part of the cycle not captured in the imaging process (almost 150 ms), such that negligible strain is expected within that period.

For validation purposes, two more data sets were acquired at UCLA (Department of Radiological Sciences) using a 1.5 Tesla scanner (Siemens Avanto, Erlangen, Germany). In one study, the subject was a normal, 35-year-old healthy male volunteer and the subject of the second study was a male patient with acute myocardial infarction (MI). Studies were approved by the institutional review board and conducted with the informed consent of the subjects. In the healthy volunteer, first a single 5 mm thick long-axis slice was acquired with meta-DENSE for processing with the proposed methods. Subsequently, two additional 5 mm slices, whose centers were separated by 8 mm, parallel to and centered about the first slice, were also acquired with meta-DENSE for processing with the conventional method. Imaging parameters, other than the number of slices, were similar for both acquisitions and only one phase was acquired for this validation study. We computed the strain from the single long axis (LA) slice using the proposed algorithm and compared it to the strain calculated from the conventional 3D method applied to the displacement field of these two latter slices.

For the patient study, which was a preliminary effort to evaluate the diagnostic value of the proposed approach, we applied our method to quantify the strain changes caused by myocardial infarction (MI). In this HIPAA compliant study, we first located the infarcted region in the myocardium of the patient using Late Gadolinium Enhancement (LGE) CMR. We next acquired DENSE images from the same single short axis slice to investigate myocardial contraction through strain mapping.

### DENSE data pre-processing

After obtaining the DENSE images, segmentation was performed by semi-automatic masking of all parts of the anatomy except for the left ventricular myocardium. Since meta-DENSE always acquires the image at a specific time from the beginning of the cardiac cycle, each image pixel corresponds to one specific myocardium voxel in all cardiac phases. Therefore, performing segmentation for only one time frame is sufficient for masking all other time steps. Using the DENSEView software [28], the 3D displacement field was generated in a matrix format to be used with our algorithm for strain calculation. From the three components of the displacement for each image pixel, it is possible to generate an image of the displacement magnitude. If this image is created at a time point that has undergone a large displacement (end systole to end diastole), it becomes a robust indicator of myocardial anatomy and hence a good template for segmentation by manual masking. It is useful to have the displacement magnitude images of a few (3-4) phases, at maximum displacement, averaged together when performing the manual masking, as it helps to avoid inaccurate masking at noisy points.

Phase unwrapping was then performed on the segmented images by scanning the myocardial area through a region-growing algorithm, while searching for sudden changes in the displacement magnitude. These changes were fixed later by adding or subtracting the correction values, which are proportional to integer multiples of 2*π *changes in phase. This step was repeated separately for all three directions of displacement and for all phases. Spatial and temporal filtering of the displacement vectors was performed at this step, as explained in *Displacement smoothing *subsection.

### Strain measurement

Using the algorithm described in the Theory section, the strain maps were then calculated from the three-dimensional displacement field for each of the myocardial material points at every time frame. To express the strain values in the local polar coordinate system, the RCL directions are required at all measurement points. In our method, these directions were calculated automatically for every single point of the myocardium. Since all deformations were calculated in comparison to the position of points at end diastole, the calculated directions at rest were used to express the strain tensor in the RCL coordinate system for all time steps.

The steps outlined in the Theory section are expected to lead to a tolerable strain calculation, such that in each local neighborhood, there exist a sufficient number of points with acceptable strains to estimate the elements of the strain tensor in that neighborhood. To void and recalculate the unreliable values within the strain tensor, strains whose magnitudes were different from the mean by more than 2 standard deviations were filtered out. Highly non-physiological strains with absolute values greater than one were eliminated before the aforementioned mean and standard deviation calculations. Next, the five pixel median filter, described in the Theory section, was applied to the strain map, in order to account for the outliers. To fill the voids created by the ignored points and to smooth the strain, a moving average filter acting over a 1 × 1 cm^2 ^area was used.

### Validation of the method

The proposed algorithm was validated first with a simulated displacement field that has its strain analytically calculated. Next, we validated our algorithm by comparison to strain calculation based on two adjacent slices. Finally, we compared our results to statistical estimations provided by Moore et al. [29] and Young et al. [30] over actual MR acquired data sets. Moore's measurement is based on 3D tagging dataset acquired for n = 31 hearts, while Young et al. has used a 3D finite element MR tagging-based model on 12 volunteers.

The simulated displacement field mimics the geometry and wall motion of LV excluding its apex. It is defined in a polar coordinate system where the *Z *direction coincides with the main long axis of the heart. The geometry of the LV before and after deformation is shown in Figure 2, with its parameters and analytical strain fully described in Appendix B. The simulated displacement field was fed into our algorithm and then the resulting values for the deformation gradient tensor, as well as the strain tensor, were compared to their analytical values. In the absence of noise, the previously described steps involving "displacement smoothing" and "filtering of the strain" were omitted to focus only on the effect of the singularity on the calculation of deformation gradient elements. To study the effect of noise on the algorithm, additive noises were simulated and independently added to each of the displacement elements. Each additive noise had a normal distribution, zero mean and varying standard deviation, which is normalized by the maximum displacement in the overall myocardial motion.

## Results

### Performance of the algorithm on simulated data

The smoothing area in the algorithm over which we minimized the quantity *L *in Equation (3) was set to 25 mm^2^. With 1 mm discretization steps, this smoothing area contained 25 data points in our simulated model and covered almost 2.8% of the cross-sectional area of the LV in the long-axis view. The resulting values at the time of maximum contraction are summarized in Tables 1 and 2.

**Table 1:** Accuracy of the elements of the deformation gradient matrix in the local coordinate system.

	shear elements						normal elements		
	
	***F***_***12***_	***F***_***13***_	***F***_***21***_	***F***_***23***_	***F***_***31***_	***F***_***32***_	***F***_***11***_**-1**	***F***_***22***_**-1**.	***F***_***33***_**-1**
Variation in Magnitude	0.006	0.113	0.320	0.013	0.179	0.068	0.381	0.192	0.152
RMS Error	0.000	0.026	0.107	0.013	0.008	0.002	0.001	0.027	0.026
Relative RMS	3.7%	23.1%	33.5%	98.2%	4.6%	2.3%	0.3%	13.9%	17.1%

The calculation was done on the simulated data for the maximum contraction (See appendix B).

**Table 2:** Error in the strain element values, corresponding to the deformation gradient tensors reported in Table 1 for simulated data at maximum contraction.

		Shear			Normal		
		
		RL	LC	CR	RR	LL	CC
	Analytical Magnitude	0.136	0.033	0	0.523	0.163	0.133
E	RMS Error	0.047	0.005	0.017	0.036	0.023	0.025
	Relative RMS	34.6%	13.8%	NA	6.8%	13.8%	18.5%

The first row of Table 1 shows the variations in the analytical values of the elements of the deformation gradient tensor from the identity matrix (***F***-***I***). The second and third rows respectively show the RMS of the error in our estimation and the relative RMS error (i.e. the RMS error normalized by the magnitude of the variation for the same element). These values were calculated for the elements of the deformation gradient tensor in the local coordinate system to avoid the propagation of the singularity condition, explained in the Theory Section, from the third column of the matrix ***F ***to its other elements. The first and second direction base vectors are nearly equivalent to the Radial and Longitudinal directions, respectively. Nearly all element values are in agreement with analytical calculations and RMS errors are less than 0.03. The only exception is the in-plane element of *F*_*21 *_which has a RMS error of 0.11, corresponding to 33.5% relative RMS error.

Table 2 shows that the RMS error for all six elements of strain is less than 0.05. In particular, the agreement of our algorithm with analytical values for those elements associated with the out-of-plane (circumferential) direction is notable. The highest error was for in-plane shear strain, *E*_*RL*_, which corresponds with the high relative error in *F*_*21*_.

Table 3 presents the effect of displacement errors in the strain calculations for the same steps reported in Tables 1 and 2 (i.e. without extra "displacement smoothing" and "filtering of the strain"). The procedure was performed for two smoothing areas of 25 and 15 mm^2^, which correspond respectively to 1/36 and 1/60 times the myocardial cross-sectional area in the long-axis direction.

**Table 3:** Effect of noise and smoothing area on strain calculation for simulated data at maximum contraction.

		Shear		Normal		
		
	Noise (σ/max displacement)	RL	LC	RR	LL	CC
Relative RMS Error in E (smoothing area = 25 mm^2^)	0	34.6%	13.8%	6.8%	13.8%	18.5%
	0.01	34.6%	15.7%	8.1%	14.1%	21.6%
	0.02	36.1%	25.5%	10.9%	15.9%	27.1%
	0.03	36.0%	32.6%	13.3%	19.0%	37.0%
	0.05	45.9%	46.9%	21.4%	23.2%	61.0%
Relative RMS Error in E (smoothing area = 15 mm2)	0	27.4%	14.4%	5.7%	9.4%	12.2%
	0.01	28.0%	21.2%	8.0%	10.3%	17.0%
	0.02	30.9%	30.7%	11.1%	14.0%	28.6%
	0.03	34.2%	42.0%	15.3%	21.1%	46.8%
	0.05	46.4%	64.7%	24.7%	28.2%	66.3%

### Comparison to the strain from two adjacent slices

**Figure 3:**
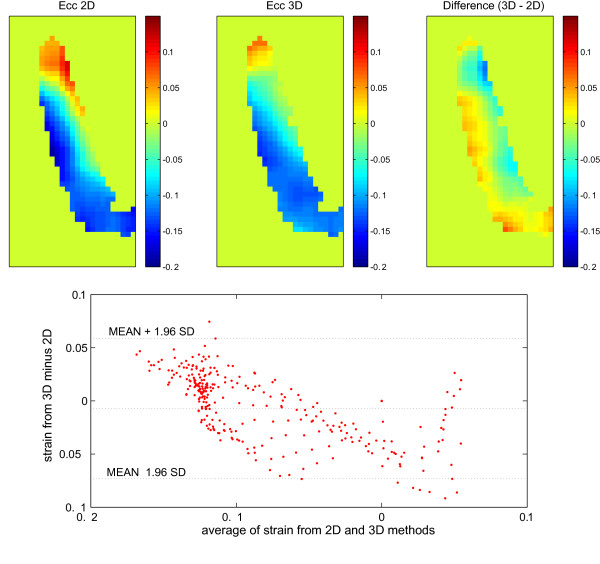
**The circumferential (out-of-plane) strain calculated from the proposed method (top left) in comparison to the same quantity calculated from two adjacent slices through a conventional method (top middle)**. The difference is shown in the top right image. The Bland-Altman plot projects the difference between these two methods in more detail (bottom). Since the measured displacement is during mid-systole, the calculated circumferential strain comprises both positive and negative values in different regions. The pattern of the strain is similar between the two methods, however, the extreme values of the strain were slightly overestimated using the proposed method.

As described in the Method section, we computed the strain from a single long-axis slice using the proposed algorithm and compared it to the strain calculated from the conventional 3D method applied to the displacement field of two adjacent slices. The circumferential strain, which represents the out-of-plane strain for LA slices, is shown in Figure 3 for both methods. The discrepancy between the two methods is shown in the third panel of Figure 3 and results in an RMS value of 0.03. A Bland-Altman plot is also added to this figure to project the difference between these two methods in more detail.

### High spatio-temporal strain measurement

**Figure 4:**
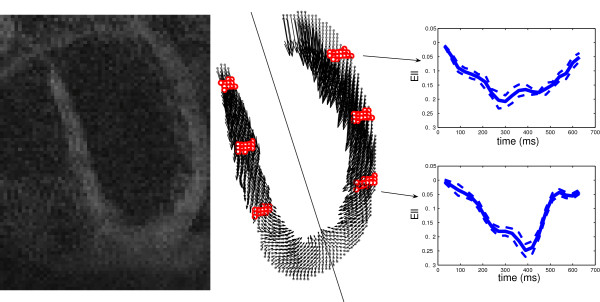
**A sample image of DENSE (left) and corresponding data acquisition points (middle) are illustrated on the left ventricle wall in the CMR long-axis slice of a healthy human heart**. The middle panel also shows the 2D projection of the motion for these material points at the time of maximum contraction (360 ms after QRS). The six regions determined with red dots are at 30%, 55% and 80% of the distance from the apex to the aortic valve in which the comparisons of Tables 4 and 5 have been performed. (Right) Mean value of E_LL _as a function of time, shown in two regions of the free wall; the solid plot lines show E_LL _averaged over the points, while the dashed lines show mean ± std over the same group of points.

DENSE images characterize the displacement field by showing the corresponding phase shift for each material point [8]. Figure 4 illustrates one sample DENSE image, together with the 2D projection of a long-axis DENSE grid (or material) point dataset acquired at the time of maximum contraction (360 ms after QRS). The points represent the positions of tissue elements at the initial acquisition time (start of systole) in the cardiac cycle.

The algorithm was applied to the acquired displacement field with a smoothing area of 20 mm^2^. The strains calculated for this dataset are compared to those measured by Moore et al. [29] and Young et al. [30]. Table 4 shows the peak values of E_LL_, E_CC _and E_RR _during the maximum systole, that is between 330 to 450 ms after the R wave, calculated in the proposed method and compared to statistical values from Moore's and Young's calculations (mean ± standard deviation intervals). This was done over six areas on the septum and the lateral (free) wall, shown in Figure 4. Points in red show 30%, 55%, and 80% of the distance from the apex to the aortic valve annulus and are called apical, equatorial, and basal. Each element of the strain matrix has been averaged in each of these six regions and for normal strains the peak values of their means are shown in Table 4. Among these 18 calculations (3 quantities in 6 anatomical regions) for this single study case, only one in-plane strain value (i.e. E_LL _of the lateral wall at the basal level) does not fall in the range of [mean ± 2σ] presented by Moore. However, this same E_LL _does in fact fall within the range of [mean ± σ] as presented by Young et al. Furthermore, in comparison to Moore's results, fourteen of our calculations out of eighteen are in the [mean ± σ] interval. Differences between our results and the results of Moore et al. and Young et al., as well as disagreements between Moore et al. and Young et al. calculations, are examined later in the Discussion section.

**Table 4:** The peak values of strain averaged over six regions on the septum and LV free wall as shown in Figure 4 compared to those reported by Moore et al. [29] and Young et al. [30].

	Region	Sepal Wall			Lateral (free) wall		
		
Strain		Present method	**Moore et al**.	**Young et al**.	Present method	**Moore et al**.	**Young et al**.
E_LL_	Basal	-0.12	-0.14 ± 0.03	-0.14 ± 0.03	-0.22	-0.15 ± 0.03	-0.19 ± 0.04
	Equatorial	-0.17	-0.15 ± 0.03	-0.15 ± 0.02	-0.18	-0.14 ± 0.04	-0.16 ± 0.03
	Apical	-0.15	-0.18 ± 0.04	-0.19 ± 0.02	-0.22	-0.19 ± 0.03	-0.18 ± 0.03

E_CC_	Basal	-0.17	-0.17 ± 0.03	-0.19 ± 0.03	-0.18	-0.21 ± 0.03	-0.21 ± 0.03
	Equatorial	-0.16	-0.16 ± 0.03	-0.20 ± 0.02	-0.17	-0.22 ± 0.03	-0.21 ± 0.02
	Apical	-0.22	-0.18 ± 0.03	-0.20 ± 0.03	-0.18	-0.24 ± 0.04	-0.22 ± 0.02

E_RR_	Basal	0.40	0.45 ± 0.12	0.21 ± 0.10	0.58	0.52 ± 0.19	0.25 ± 0.09
	Equatorial	0.52	0.42 ± 0.19	0.16 ± 0.10	0.55	0.38 ± 0.18	0.21 ± 0.10
	Apical	0.55	0.36 ± 0.22	0.07 ± 0.06	0.64	0.49 ± 0.29	0.10 ± 0.06

Table 5 shows the shear strains for the same six regions at the time of maximum deformation, i.e. from 330 to 450 ms after the R wave, compared to Moore's calculations. Comparing the results of our single study to those of Moore et al., four out of eighteen calculated shear strains do not fall in the range of [mean ± 2σ] presented by Moore et al., of which three relate to E_RC_.

**Table 5:** The peak values of shear strain averaged over six regions on the septum and LV free wall as shown in Figure 4, at the time of maximum deformation, compared to those reported by Moore et al.

Strain	Region	Sepal Wall			Lateral (free) wall		
		
		Present method	**Moore et al**.	Variation coef. (%)	Present method	**Moore et al**.	Variation coef. (%)
E_LC_	Basal	0.02	0.01 ± 0.05	500	-0.03	0.03 ± 0.05	166
	Equatorial	0.03	0.03 ± 0.05	160	0.05	0.03 ± 0.04	133
	Apical	-0.03	0.04 ± 0.05	125	0.02	0.04 ± 0.03	75

E_LR_	Basal	-0.06	0.03 ± 0.09	300	-0.11	0.05 ± 0.07	140
	Equatorial	-0.05	0.08 ± 0.05	62.5	0.10	0.07 ± 0.06	85.7
	Apical	0.09	0.06 ± 0.09	150	-0.09	0.05 ± 0.08	160

E_RC_	Basal	0.12	0.01 ± 0.06	600	0.04	0.05 ± 0.07	140
	Equatorial	0.03	0.02 ± 0.08	400	0.19	0.05 ± 0.06	120
	Apical	-0.20	0.11 ± 0.10	91	0.26	0.05 ± 0.08	160

**Figure 5:**
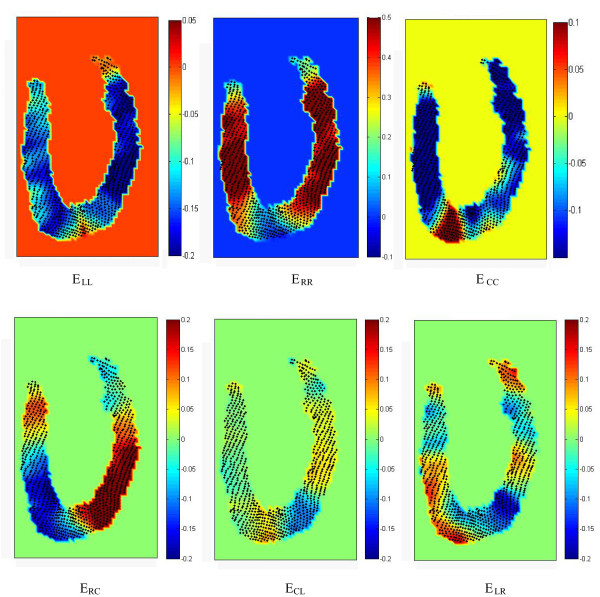
**Distribution of all normal (top row) and shear elements (bottom row) of the strain matrix in the polar coordinate system at peak systole (t = 360 ms)**. Strains were calculated from a single slice displacement data, using the proposed method. The results in the region very close to the apex suffer from the fact that RCL directions are ill-defined there. The connection of the right and left ventricles may also cause some deviations from the normal strain in this particular region. The contraction in the apical lateral wall is not in its maximum at this phase (t = 360 ms) as it is also evident from Figure 4. This figure shows that normal strains, in contrast to shear strains, are almost uniform for the rest of the lateral and septal wall at this time point.

**Figure 6:**
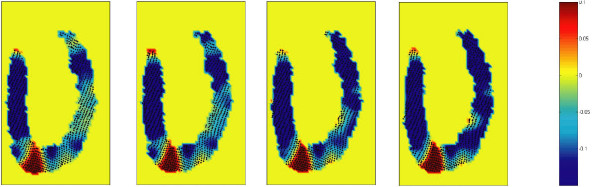
**Map of out-of-plane strain (E_cc_) close to maximum systole (from 300 to 390 ms) (4 frames are shown here)**. The first image (left) corresponds to the t = 300 ms (after R wave) and with a time step of 30 ms, the last one shows the strain at t = 390 ms. The movie available in the online supporting materials (Additional file 1) helps readers to clearly perceive the results of the method for an extended period of time.

Figure 5 shows the distribution of all six elements of strain at peak systole (t = 360 ms) and Figure 6 shows the map of out-of-plane strain (E_CC_) close to maximum systole (from 300 to 390 ms), i.e. 4 frames in total. The movie available in the online supporting materials (Additional file 1) helps readers clearly observe the results of the method for an extended period of time.

### Patient study guided by LGE

**Figure 7:**
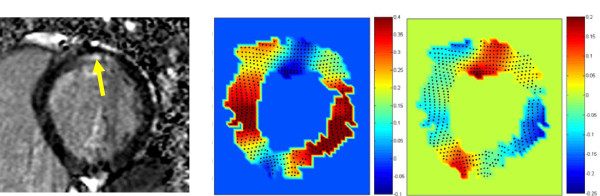
**The myocardial infarction region is revealed through the elevated signal intensity of LGE at the subendocardium of the basal-posterior part of the heart (yellow arrow, left)**. The calculated radial (middle) and longitudinal (normal to the plane, right) strains show a clear strain difference between the infarcted area and its neighboring regions. This figure shows that the proposed method is able to reveal the impairment caused by infarction in the through-plane strain. As expected, the lack of contraction in one region extends beyond its boundary through its mutual connection to the rest of the continuum.

In the clinical study described in the Methods section, the infarcted region in the myocardium of the patient with acute MI was located through LGE CMR. Figure 7-a reveals the MI region through the elevated signal intensity at the sub-endocardium of the basal-posterior part of the heart (yellow arrow). Application of our method to the displacement field acquired from meta-DENSE images of the same single short axis slice resulted in the strain maps in the middle and right maps of Figure 7, which present the radial (in-plane) and longitudinal (normal to the plane) strains, respectively. In both images, the infracted region, in contrast to its neighboring regions, shows a clear strain difference with the expected values of strains for the normal regions.

## Discussion

Herein, we proposed an analytical method for strain calculation from a single slice of displacement data. The planar nature of the data points imposes a near-singularity condition when calculating gradients in the direction normal to the surface, which makes these gradients highly sensitive to noise. Although meta-DENSE measures the three-dimensional displacement field from a Lagrangian curved reference surface, rather than an Eulerian flat reference plane, the non-planar configuration of the data is generally insufficient to overcome the effect of singularity. We approached this problem by assuming some physical/mathematical constraints that are shown to lead to an improvement in the accuracy of the strain calculation, such that the points with acceptable strain are abundant enough to be used for the correction of strains in other points of the LV myocardium. In other words, in each local neighborhood, we only need a sufficient number of points with acceptable strains to estimate the elements of the strain tensor in that neighborhood.

The proposed method is a multi-step process comprised of median filtering of the displacement to application of the incompressibility and temporal filtering of the calculated strain. This results in different degrees of accuracy in the estimation of each element of the deformation and strain tensors. Some parameters, such as the smoothing area or size of median filter, may depend on the acquired data characteristics like SNR and resolution. In particular, the size of the median filter was chosen as minimum in the beginning. The overall outcome for the acquired data demonstrated that there is no need to increase the size of the median filter, since this filter along with the other applied methods, described above, provides sufficient smoothness to achieve reliable calculation for in-plane components of the deformation gradient tensor.

### Simulated data

Verifying the algorithm's precision over analytically simulated data reveals the accuracy level for each step of the algorithm, as well as for the corresponding deformation elements. Moreover, testing the algorithm on the simulated data helps to investigate the effect of restraining factors, such as noise, on the accuracy of the calculated strain through the suggested procedure. Simulation is also important because there is no gold standard for strain estimation based on other computational methods. Although the methods that use multi-slice imaging can provide more accurate information about out-of-plane deformation, the large slice thickness and poor resolution in the out-of-plane direction, the curvature of the LV wall, and finally the inherent noise in the acquisition process substantially decrease the accuracy in the estimation of the shear and normal strains associated with that direction. Therefore, the validity of the overall algorithm has been verified in two ways: comparison of the results to the analytical strain available for a simulated displacement data and the calculated strain of a conventional two-slice acquisition.

The algorithm performed accurately for the simulated data with RMS errors of less than 0.03 for all elements of deformation gradient tensor except for *F*_*21*_. The RMS error of 0.11, corresponding to 33.5% relative RMS error, for this in-plane element of deformation gradient tensor is related to the filtering of its high spatial changes. This problem does not pertain to our method and remains the same for 3D methods that take advantage of more than one imaging slice depending on the smoothing required. Excluding the *F*_*21 *_error, RMS errors for elements in the third column of matrix ***F ***were greater than the average error of other two columns, as expected. The error, however, was not as high in the third column as the singularity condition would cause in the absence of the applied constraints. In terms of relative error, *F*_*23 *_had a large relative RMS error of almost 100% despite the small absolute error of 0.01. This was caused by the fact that *F*_*23 *_was nearly zero in our simulated deformation. This problem was more pronounced in the calculation of *E*_*RC*_, which was exactly zero in our simulation. Therefore, the small RMS error of 0.017 in *E*_*RC *_was translated to an un-bounded relative error for this component of strain.

### Smoothing area and noise

The main reason for the relatively large error in calculated *F*_*21 *_was the sharp spatial changes of this element in the radial direction (Figure 2, lower right panel), prescribed by our simulation. Calculation of this element over an extended neighborhood smoothed these fast non-physiological changes and resulted in considerable relative RMS difference. Nevertheless, the global map of this quantity as well as its maximum and minimum was predicted well as shown in Figure 2, lower right panel. By decreasing the smoothing area from 25 mm^2 ^to 15 mm^2 ^in Equation 3, the RMS and relative RMS errors of this matrix element have dropped to 0.08 and 26.1%, respectively. This reduction of the smoothing area also decreased the 34.6% relative RMS error of *E*_*RL *_to 27.4%. In general, the smoothing in displacement and strain decreases the sensitivity of the calculation to sharp changes of the myocardial behavior. We cannot, however, decrease the smoothing area regardless of the noise level, since the extent of the smoothing area is proportional to the noise reduction, as shown in Table 3.

For the in-plane elements of strain, Table 3 shows that adding noise with σ = 0.01 times the maximum displacement does not considerably change the error for the larger smoothing area, since the effect of smoothing over a neighborhood is dominant. Although the relative RMS error is less for a smaller smoothing area at low noise, for noise with (σ/maximum displacement) equal or greater than 0.02, the error is less for the larger smoothing area. The optimum smoothing area depends on the noise level in the images as it was set to 20 mm^2 ^for the in vivo acquired data.

The relative RMS error for in-plane strains increases almost linearly with the noise standard deviation, but in a quadratic fashion for strain elements associated with the out-of-plane direction. This illustrates the higher sensitivity of these elements to noise, as explained in the Theory section.

### Comparison to the strain from two adjacent slices

In the second in-vivo study the proposed method was compared to the conventional two-slice method. The measured displacement was during mid-systole; therefore the calculated circumferential strain consisted of both positive and negative values in different regions. Figure 3 demonstrates that the outcome of the proposed method is in close agreement with the results of the standard 3D method that requires the acquisition of two adjacent slices. The pattern of the strain is similar between the two methods; however, the extreme values of the strain were slightly overestimated using our proposed method. This difference can be partially explained by the effect of curvature on the strain calculation in the common 3D method. That is, the shortening and lengthening are underestimated along the chord compared to their values on the curve itself. Furthermore, it should be noted that the wall curvature erodes the actual area for which we calculate the strain from adjacent slices. We compared the results of our proposed and standard 3D methods with analytical solutions for our simulated displacement field and verified that the discrepancies are partially derived from the aforementioned shortcomings of the 3D method. Nevertheless, discrepancies can be also related to the "singularity condition" of the proposed method, which was explained in the Theory section.

Overall, it seems that our methods, which have been validated through both the two-slice and the simulated displacement data, yield an acceptable estimation of the strain for in- and out-of-plane strains from the experimental meta-DENSE data.

### Comparative study

To further validate our results, we compared them to those of Moore et al. [29] and Young et al. [30]. Moore et al. estimated wall thickening to be 0.36 to 0.52, which is more consistent with available physiological data (60% ejection fraction) and clinical evidences [31,32].

Compared to Moore et al., 17 of our 18 strain calculations were within their range of [mean ± 2σ] and 14 were in the [mean ± σ] interval. One possible reason for the observed discrepancy is the averaging of the strains over regions of the myocardial wall. For example, the peak value of apical E_CC _on the lateral free wall, calculated by our method is -0.18 in Table 4; however, if we only focus on the epicardium, this value becomes -0.20, which no longer falls out of [mean ± σ] range.

Lessick et al. [31] observed increasing gradients of thickening from base to apex. Our method shows this clearly on the entire sepal wall as well as on most of the free wall through the increase in E_RR_, whereas Moore's results do not show this pattern on either wall.

For shear strains, the agreement of our results with Moore's is acceptable, although a little less so for normal strains. (5 were out of the [mean ± 2σ] interval.) Note, however, that the variation coefficients (σ/mean) of shear strains are in the range of 62.5% to 600% (average: 200%) in Moore's calculations. These large variations indicate either a high inter-subject variability or a large measurement error margin.

It is worth noting that three of the five largest discrepancies, i.e. calculations out of the [mean ± 2σ] interval, relate to E_RC_. A closer look at the data set revealed a substantial asymmetric torsional movement from the equatorial to the apical level of the heart in this particular subject. This movement results in a significant positive E_RC _in the lateral free wall and negative E_RC _for the sepal wall close to apex. Thus, our method detected values which may not fall in the range given by Moore et al.

Besides the peak strain values, reported in the previous studies, the temporal changes of the strain components may also be important as they can reveal additional details of cardiac function. For instance, the mean value of E_LL _as a function of time is shown in two regions of the free wall in Figure 4. It illustrates that although the contraction profiles are almost similar in the base and apex in the first 250 ms of the cycle, the peak strain at the apex happens at a later time (400 ms) and the strain decreases in magnitude rapidly afterwards. The importance of the temporal changes further justifies our proposed algorithm (strain calculation from a single slice), which saves time on the number of acquired slices. The saved time can be utilized to improve the temporal resolution.

### Clinical study

The diagnostic potential of this non-invasive method in localization of the hypo-kinetic regions of the myocardium was shown by comparing it to LGE CMR. The comparison was performed in a short axis imaging plane without any assumption on the symmetry of torsional motion of the heart. The abnormal strain is seen in a wider section around the MI region as expected in a continuum, i.e. the lack of contraction in one region extends beyond its boundary through its mutual connection to the rest of the continuum [33]. For this reason, strain measurement is not supposed to replace the LGE. It is, however, important to note that the strain map is quite useful for infarct quantification in terms of its effect on the myocardial contractility and eventually the heart function. Figure 7 shows that the proposed method is able to reveal the impairment caused by infarction in the through-plane strain. There is another small area in the anterior part of the heart with unexpected strain values for radial and longitudinal strains. This is the location where the right and left ventricles are connected and thus we may expect some deviation from the normal strain in this particular region.

### Limitations of the technique

The singularity condition, explained in the Theory section, poses an instability problem for the proposed method if the noise level is high. In general, the acquisition of the high spatio-temporal displacement fields with sufficient SNR is a non-trivial process and therefore data of sufficient quality for the proposed method may be difficult to obtain routinely. This highlights the value of the methods that help to extract as much information as possible from the acquired data.

A source of error in our results could be that we assume the heart function is completely periodic and hemodynamically stable during relatively long acquisition times. This assumption may not be sufficiently accurate and could result in errors.

Finally, it should be noted that the results in the region very close to the apex suffer from the fact that RCL directions are ill-defined there. This imposes some problems when the strain values are transformed to the polar coordinate system.

## Conclusion

The proposed method is able to calculate all components of the 3D Lagrangian strain tensor for systolic and diastolic phases in the left ventricle from a single-slice of 3D displacement data by assuming incompressibility of the myocardium as a physical constraint. The feasibility and the validity of the calculation of all six elements of the myocardial strain tensor from a single-slice of 3D displacements has been demonstrated. The feasibility of obtaining very favorable spatio-temporal resolution for tissue tracking by DENSE CMR from human subjects has also been shown. Furthermore, the clinical potential of the method for the evaluation of myocardial damage was demonstrated through the comparison with LGE CMR.

The method developed in this study is general and can be used with other data acquisition techniques such as zHARP or slice following 3D cine DENSE. Our work provides a framework for the detailed analysis of cardiac function. For example, it is able to demonstrate the asymmetry of strains and non-uniform contraction patterns within the ventricular wall. It is also able to calculate 3D strains from a reduced dataset, using some assumptions, which helps reduce the total scan time relative to a normal examination.

## Competing interests

The authors declare that they have no competing interests.

## Authors' contributions

ANM developed the method; acquired and processed the data; and drafted and finalized the manuscript. NRS conceived of the original study (along with HW and MG) and participated in drafting and finalizing the manuscript. HW prepared and updated the DENSE sequence for this study. MG, DBE and JPF participated in the study's design and coordination. All authors read and approved the final manuscript.

### Appendix A: *Error Analysis*

The expected error in each component of the 3D displacement measurement is assumed to be similar, since the displacement encoding sensitivity is comparable for these directions. Considering the average separation of points in each of these three directions, we expect an error of the similar order for the first two columns of the ***F ***and ***G ***tensors (δ_1_), but a substantially greater error for the third column (δ_2_). Using the suggested method for the calculation of *F*_*33 *_(Equation 4), the expected error will decrease to 2 δ_1_:(A-1)

This approximation is derived from the fact that for a realistic deformation of the myocardium, values of diagonal elements of matrices ***F ***and ***G ***are perturbations around unity, while the other matrix elements are minimally perturbed around zero. Equation (5), however, results in a similar expected error (δ_2_):(A-2)

Nevertheless, that equation still can help in the estimation of ***F***, considering the fact that errors in the elements of the third column are biased towards larger absolute values for those elements. This will in turn result in a compounded overestimation of the absolute values of *E_13 _*and *E_23 _*strains, as they have a quadratic dependence on ***F***. Hence, we calculate the sum of these two latter quantities based on ***F ***and ***G ***separately. We substitute elements of ***F ***by using Equation (5), only if the calculated sum of *E_13 _*and *E_23 _*based on ***G ***is smaller than that based on ***F***.

### Appendix B. *Simulated displacement field for the LV*

A simulated displacement field is suggested in a polar coordinate system where the *Z *direction coincides with the main long axis of the heart. The geometry of the LV is shown in Figure 2 with its parameters determined by the following equations:(B-1)

where *R*_1 _and *R*_2 _represent the radius of the endocardium and epicardium respectively, as a function of longitudinal position (*Z*). Also, *R*_0 _and *L *show the maximum radius and length of the simulated part of the LV epicardium.

For simplicity, the displacement is considered axisymmetric (∂/∂φ = 0) and may be divided into two components: in-plane deformation and axial rotation. The feasibility of such a decomposition can be simply perceived by transforming all material points in the distorted configuration back to the original plane (e. g. , by setting *φ *to 0 for all points) in order to consider the in-plane deformation first and then apply the rotation as the second step of the deformation process. Due to axial symmetry, we know that returning points to their original plane does not result in non-physical conditions such as overlap in the in-plane deformation. Hence, there will be no problem in determining two separate deformation tensors for these two steps. The total deformation tensor is the multiplication of these two tensors:(B-2)

The in-plane displacement field is defined below:(B-3)

where *f*(t) is a function of time that specifies the level of contraction during the cardiac cycle and *g*(*R*) is an arbitrary function of radial distance *R*. The maximum value of *f*, which corresponds to maximum contraction in the LV, is chosen to be 0.4 so as to make the displacement field match with physiological data, in which a 40% thickening of the LV wall is generally observed in the radial direction at maximum contraction, while the contraction in the circumferential and longitudinal directions have averages around 15% and 20% respectively [34]. If *g(R)*is replaced by any arbitrary function of *R*, this displacement field will still satisfy the incompressibility condition. The deformation tensor for this displacement field is calculated analytically in the Cartesian coordinate system as follows:(B-4)

The matrix above has a determinant of unity, which guarantees tissue incompressibility during this deformation. (*f*(t) is shown as *f *for simplicity.) To improve the realism of the resultant deformation, we used *g*(*t*) = 2*f*(*t*)(*R*-*L*/5), where *L *is the length of the LV at rest. This mimics the longitudinal movement of the base.

The axial rotation around the main long axis of the LV is shown by an angle which is a function of *R*, *Z *and time, *i.e*. *β*(*R*, *Z*, *t*). This rotation corresponds to the following deformation tensor:(B-5)

It can be shown that det(*F*_*Rotation*_) = 1. For simplicity, we assumed that *β *depends on time as a separable variable. For this simulation, we chose the temporal changes of *β *to be in the general form of *f*(t):(B-6)

Since the heart's torsion occurs ahead of its shortening, *t*_*0 *_is added to the argument of function *f*(t) in Equation (B-5). Moreover, we assume *β *to be a linear function of *Z *that generates a 15-degree rotation of the ventricle from base to apex, which corresponds to physiological ventricular torsion [35].

Once we calculate the ***F***_*total *_through Equation (B-2), the analytical strain tensor can be calculated exactly from Equation (2). This analytical strain is used as the ground truth to validate the proposed method when it is applied to the aforementioned simulated displacement. To evaluate the stability of the method, errors were simulated as additive noises and independently added to each component of the displacement field . Each additive noise has a normal distribution, zero mean and varying standard deviation which is normalized by the maximum displacement in the overall myocardial motion. (See Table 3).

## Supplementary Material

Additional file 1 **Strain mapping results from single slice DENSE data**. This movie demonstrates the results of the proposed strain calculation method for an extended period of time. It displays the high spatio-temporal resolution maps for all six elements of the strain tensor and helps readers clearly observe how the local Radial, Circumferential, and Longitudinal (RCL) directions are defined for all myocardial points on a single slice of the LV in the long axis view. Click here for file
